# How should researchers cope with the ethical demands of discovering research misconduct? Going beyond reporting and whistleblowing

**DOI:** 10.1186/s40504-020-00102-6

**Published:** 2020-08-06

**Authors:** Knut Jørgen Vie

**Affiliations:** Work Research Institute – Oslo Metropolitan University, Stensberggt. 26, Oslo, Norway

## Abstract

In this paper, I will argue that making it mandatory to report research misconduct is too demanding, as this kind of intervention can at times be self-destructive for the researcher reporting the misconduct. I will also argue that posing the question as a binary dilemma masks important ethical aspects of such situations. In situations that are too demanding for individual researchers to rectify through reporting, there can be other forms of social control available. I will argue that researchers should explore these. Finally, framing the issue as a question about the responsibilities of individual researchers masks the responsibilities of research institutions. Until institutions introduce measures that make this safe and effective, we should not consider reporting research misconduct mandatory. I will discuss this in light of both quantitative and qualitative data gathered as part of a survey in the PRINTEGER-project.

## Introduction

Self-regulation is fundamental to research. Robert Merton listed *organized skepticism* as one of the norms of his *scientific ethos* (Merton 1973), and claimed that the mutual scrutiny of researchers is what justifies giving them autonomy and trust. By criticizing each other’s work, researchers are driving research forward, improving both its results and methods. *Research integrity* emerged as an academic field with the realization that researchers sometimes engage fraudulent practices (Steneck 2006) like falsifying and fabricating data (Fanelli 2009), along with other forms of misconduct often labeled *questionable research practices* in the literature (Shamoo and Resnik 2015). Self-regulation took on a new role in this field, as most of the misconduct cases we know about were exposed and reported by colleagues or collaborators of those who engaged in it (Ben-Yehuda and Oliver-Lumerman 2017). If we do not discover and punish misconduct there will be no risk in engaging in it and our trust in research results could diminish. Self-regulation is therefore essential to ensuring that research is trustworthy.

**Table 1:** Qualitative questions

1	How did you first learn about the instance of research misconduct?
2	Please describe the specific instance of research misconduct
3	What did you do when you became aware of it?
4	Whom (titles only) did you talk with about the research misconduct? How did you feel during this experience?
5	Were you able to talk with the individual(s) who were involved about it? Please describe your interaction.
6	Was the instance reported? If so, to whom and by whom? Whether the decision was to report or not, how was the decision made? What were the factors underlying the decision to make the report?
7	What was the outcome? How did you feel about the way it was handled?
8	Did you think anything changed as a result?
9	Is there anything you would have done differently?

**Table 2:** “I feel confident that I would be protected as a whistleblower” (*N* = 1126)

To a large degree	10,5%
To a certain degree	30,3%
To a small degree	31%
Not at all	28,1%
	100%

**Table 3:** “I feel confident that the faculty (or other relevant bodies in the university) would take seriously the whistleblowing and act accordingly” (N = 1126)

To a large degree	23,4%
To a certain degree	39,3%
To a small degree	23,9%
Not at all	13,3%
	100%

**Table 4:** Outcomes and characteristic responses

	Characteristic responses	Number of cases (*N*=)	Whistleblowing/Reporting
Non-action	“No, I can’t because of hierarchy. It is a superior and denouncing could have a negative impact on my job”, “no idea [where] I can report this”	24	N/A
Action (total)		124	
Success	“I feel really good about the way it was handled: the swift response of the committee; the way in which the member of the committee helped me walk through the options of what to do etc.”, “The person got fired […] the whole field changed its way of conducting research and dealing with data”	30	25
Failure	“Was told everyone should be on all papers […] would have risked career if I had been more vocal”, “The department head covered the person who plagiarised. […] I was told the case was not serious enough because the work had not been published”	58	27
Ambiguous	“paper was rejected; no clear consequences”, “the results in the submitted publication were changed; the whistleblower was very disappointed that there were no sanctions for the [project leader]”	36	27

The question has therefore been raised whether reporting misconduct should be considered a duty among researchers (Malek 2010), and whether it should be mandatory. Satalkar and Shaw recently argued that *raising a concern* when discovering misconduct “at the earliest moment, to the appropriate level, in a collegial constructive fashion and in the spirit of improving science and research” (2018, 336) should be formalized into codes of conduct. They see their suggestion as one of several measures needed for building a culture for self-regulation and add that we also need stronger protections for those who report misconduct. They write that researchers should report any deviation from standard practices, and that this should lead to an impartial investigation.^1^

Almost all the researchers they interviewed expressed that not raising a concern compromises one’s integrity as a researcher. Among the responses, the primary argument they identify is that reluctance to raise a concern when discovering misconduct has *negative consequences*; it corrupts the academic and scientific culture, harms patients in the long run and wastes resources (Satalkar and Shaw 2018).

Satalkar and Shaw point out that the American system provides one of the strongest formulations of the duty to report misconduct, while European guidelines tend to treat reporting misconduct as voluntary. The U.S. National Academy of Science states that despite the difficulties involved with reporting misconduct, “someone who witnesses a colleague engaging in research misconduct has an unmistakable obligation to act” (National Academy of Sciences 2009, 19). Acting here means reporting the misconduct to the relevant authorities, according to federally mandated institutional policies and procedures. This demand is also justified with reference to the consequences of not reporting misconduct, as this,

has the potential to weaken the self-regulation of science, shake public confidence in the integrity of science, and forfeit the potential benefits of research. The scientific community, society, and the personal integrity of individuals all emerge stronger from efforts to uphold the fundamental values on which science is based (National Academy of Sciences 2009, 19).

Maintaining trust in science and its integrity is an important consequence of reporting misconduct, and these consequences outweigh the discomfort and risk of reporting, the argument goes.

In response to this, I will argue that a consequentialist approach leads to a conclusion that formalizing the duty to report misconduct into codes of conduct is too demanding on researchers. Using data gathered as part of a survey in the PRINTEGER-project^2^ I will show that researchers can find themselves in situations where they discover misconduct and experience that reporting it is both very risky and unlikely to be successful. Based on data indicating that this risk perception is reasonable, I will argue that it would be a mistake to make it mandatory for researchers to make the case known when discovering misconduct.

My approach in proposing this can be described as a form of *empirical ethics* in the sense that it a “provision of facts important for normative arguments” (Salloch et al. 2015, 6). Data about how researchers perceive and suffer negative consequences concerning reporting misconduct is relevant for the question whether such negative consequences are outweighed by the potential positive ones.

I will also present data in order to show that researchers have a broader spectrum of options available to them than just reporting when they discover misconduct. Malek (2010) argues from a theoretical position that such alternatives are relevant for the discussion of researchers’ duties, and the data gives some insight into what these alternatives are. When reporting is too demanding there can be other approaches to handle the situation. Moreover, researchers can use their experiences to prevent further misconduct.

In the literature, the reporting of misconduct is the most common approach to discussing self-regulation of research misconduct (Bouter and Hendrix 2017; Faunce et al. 2004; Lubalin and Matheson 1999; McIntosh et al. 2019; Mecca et al. 2014; Redman and Caplan 2015). The phenomenon is also called whistleblowing, which can be understood as disclosing knowledge about misbehavior as an organizational member (or former member), to somebody who can take action (Near and Miceli 1985). This includes actors both within the organizations where the misconduct took place and outside it to institutions like the media or research ethical committees. While these studies give us some insight into self-regulation, they do not give us the full picture.

We therefore need a broader theoretical lens. *Social control theory* offers such a lens (Ben-Yehuda 1986; Fox and Braxton 1994; Hackett 1994; Vaidyanathan et al. 2016). One way of defining wrongdoing within this approach is to say that it is “any behavior labeled as wrongful by social control agents” (Palmer 2012, 243). A *social control agent* is somebody who “represents a collectivity and that can impose sanctions on that collectivity’s behalf” (Greve et al. 2010, 56). Defining wrongdoing in relation to what social control agents do operationalizes the concept and makes it possible to explore it empirically, by studying the actions of these agents. Importantly, it also allows us to identify and discuss other kinds of control behavior than whistleblowing.

Ben-Yehuda and Oliver-Lumerman (2017) have applied the concept of social control agents in the context of research integrity. They claim that individual researchers act as important social control agents and that they are socialized into this role during their training. The definitions of research integrity and research misconduct are contested (Shaw 2018) and has different meanings in different countries and in different research cultures. In addition, researchers often experience ambiguity in research ethical and research integrity questions (Johnson and Ecklund 2016), in the sense that it is not always clear to them what the right thing to do is. By defining misconduct as what the respondents themselves label as misconduct, we avoid having to force their responses through theoretical definitions of integrity and misconduct, which they do not necessarily recognize.

Examples of social control in this context can be things like direct confrontation with the perceived wrongdoer, or informal exclusion from further research projects. Social control will be the main theoretical lens for analyzing the data in this paper. While this theory is descriptive and not normative, it does normative work as a form of empirical ethics, through providing “a fuller understanding of a moral phenomenon” (Salloch et al. 2015, 6), giving an overview of how researchers can react to discovering misconduct. Theoretical discussions of ethical situations, like those that can emerge when discovering research misconduct, risk missing ethically relevant aspects of such situations, as they are abstracted away from their context (Musschenga 2005). This risk can be mitigated by empirical research on how such situations are actually perceived and handled by those who find themselves in them.

This paper therefore has two purposes. Firstly, it aims to present challenges associated with whistleblowing in research, supplemented by descriptions of with alternative forms of social control. Secondly, it aims to contribute to the discussion about the duties researchers have when discovering misconduct in light of these findings. The combination of empirical findings with normative approaches has contributed fruitfully to informing ethical questions in other fields, like for example euthanasia and informed consent (Parker 2009), finding ethically relevant aspects through an empirical approach. In this paper, as we shall see, the data reveals that researchers have several ethically relevant options when considering what to do when discovering misconduct, and that these options are masked when the issue is framed as a matter of blowing the whistle or not. My aim is not to just to argue, but also to *show* that researchers’ duties when discovering misconduct should be discussed in a broader perspective.

## Methodology and data

The data was gathered as a part of a web-based survey conducted by the PRINTEGER project.^3^ The quantitative results have been summarized in a deliverable in the project (Mamelund et al. 2018),^4^ which gives further information on the survey design and composition of the sample. In addition to 46 closed-choice questions, it contained nine open-ended questions about the participants’ personal experiences with research misconduct, if any.

Recruitment was done by emails, which were forwarded to all researchers at the eight, institutions participating in the PRINTEGER project, except for the PhD-students at one of the institutions, to whom we did not get access. The survey was approved by the leadership at the participating institutions, and by the relevant research ethics and data protection authorities in the partner countries. The respondents were asked whether they consented to participate in the survey. The survey resulted in 1211 participants, from a population of 20,815. There was however some attrition due to some participants declining to continue with the survey after having read the consent form (*n* = 79) and some participants leaving the survey unanswered (*n* = 6). This leaves us with a net sample of 1126 respondents, and a net response rate of 5.4%. Of the 1126 respondents, 192 had knowledge about specific misconduct cases and answered the open-ended qualitative questions.

The qualitative questions were adapted from the validated and revised Scientific Misconduct Questionnaire (SMQ-R) (Broome et al. 2005; Habermann et al. 2010): Table 1.

In addition to the qualitative questions, two of the quantitative questions are relevant for the analysis. The respondents were asked how confident they are that they will be protected as whistleblowers, and how confident they are that the relevant authorities will take whistleblowing seriously and act accordingly (see the figures in the results section for the full formulation of these questions).

The data has some limits; in cases of perceived misconduct there is more than one side. In this paper however, the responses were taken in good faith, as the theoretical framework defines misconduct as what social control agents label as such. Viewed from other theoretical positions however, it is possible that the respondents gave a skewed representation of the cases, in order to put themselves in a favorable light, which is a common response bias when it comes to ethical questions (Randall and Fernandes 1991). False accusations of misconduct also happen, and there is no way to determine if there were such cases in the dataset. Another limit is the way in which the responses were gathered. Most of the responses were short, possibly because they were part of a long survey. It is therefore possible, that important details were not included in the responses. Some of the quotes included in this paper had spelling mistakes. These were corrected, and these corrections are marked with brackets.

Another important limit is the fact that the survey only reached respondents who are currently working as researchers, or at least still have access to their institutional email addresses. A common worry in the data is the potential risk reporting misconduct involves for one’s career, and the survey is not suited to catch such consequences. A final limitation is the very low response rate. This does not diminish the value of the qualitative data when it comes to making the normative points this paper aims to make. It does however mean that the quantitative data has limited utility. The normative discussion in this paper therefore includes supplementary references to studies that point in the same direction.^5^

## Results and analysis

The quantitative part of the survey included questions about whistleblowing that give us a starting point for discussing the qualitative answers. While whistleblowing is only a special form of social control, and only gives a limited perspective on what I am interested in in this paper, the researchers’ responses to these questions give us an indication of their level of trust in their respective institutions. [Tables 2 & 3].

Here we see that relatively few researchers replied that they have confidence in their institutions when it comes to protecting whistleblowers and rectifying the situation in misconduct cases. More than half answered that they have low confidence or no confidence at all that they will be protected as whistleblowers. The situation is somewhat better when it comes to researchers’ beliefs about whether their institutions will take whistleblowing seriously. Nevertheless, around a third of the respondents reported low confidence or no confidence at all that they would be taken seriously.

The 192 responses to the qualitative open-ended questions were coded in NVIVO by the author, and were analyzed thematically (Creswell 2014). Initially, a simple coding scheme was used, where the cases were sorted into two categories, based on whether or not the researcher decided to take action when faced with a case of perceived misconduct or breach of integrity. 44 of the responses fell outside of this scope.^6^ This leaves a case sample of 148.

Delineating between action and non-action was more complicated than expected. Social control can differ in intensity and effort from case to case. In its mildest form, simply discussing the case informally with an external confidant can constitute taking action against the perceived transgressor, as it has the potential to hurt his or her reputation. In this paper however, the threshold was set somewhat higher, in line with the theoretical perspectives on social control agents. The cases were not coded in the action category unless there was some form of attempt to rectify the situation and label the misconduct.

In 24 of the 148 cases, the respondent decided to take no action according to these criteria, thus dropping out of the process by not mobilizing enough motivation to take action. The most prevalent reason for not taking action was fear of personal cost (*n* = 12). The 124 remaining respondents deciding to take some action. Taking action however is not enough when it comes to successfully exerting control; it is still possible to fail. The responses in the action category were coded in two further categories, success and failure, where the successful cases had the characteristics that they ended with consequences for the norm-breaker, and any damage caused by the misconduct was rectified, like for example data theft or denial of authorship by collaborator or senior researcher.

Most of these cases were failures (*n* = 58), and the primary characteristic of the responses in this category, which was present in all of them, was the impression that there were no consequences for the perceived transgressor. In some of the cases, the respondents also stated that there were personal costs or threats thereof to either the respondents themselves or another party involved in the attempted control. This took two different forms. 1. Some experienced costs related to lack of rectification (*n* = 28). This means that there was some victim of the misconduct. Because they failed to solve the situation, the losses related to the misconduct were not rectified. 2. There were also costs or threats related to the attempt at exerting control (*n* = 35). In other words, trying to solve the situation had negative personal consequences. In 25 of these cases, both these issues were present.

A third theme emerged during this phase of the coding. Many of the cases did not fit neatly in the success or failure categories and were therefore coded as *ambiguous* (*n* = 36). These cases were ambiguous for different reasons. Most of the respondents felt that although the perceived wrongdoer suffered some consequence due to his or her actions, the punishment or other reaction was not proportionate with the seriousness of the case (*n* = 31). While the perceived wrongdoer suffered some consequence, like rejection of attempted publication, attempts to pursue the case further did not lead to what the respondents would consider appropriate sanctions. More seriously, some of the cases were ambiguous in part because either the respondent or another innocent party suffered or felt threatened with significant personal consequences (*n* = 11). In some of the cases, both these concerns were present. The remaining 30 cases were coded as successful without significant ambiguity.

In sum, the largest category where the respondent decided to take action was the failure category (*n* = 58). The second largest was the ambiguous category (n = 36). This made the success category the smallest of the three (*n* = 30). This gives us 30 cases of success, and 118 cases of non-action, failed attempts at exerting control or ambiguous results. This means that 20% of the cases resulted in an unambiguously successful exercise of social control from the perspective of the respondent.

Finally, the cases were coded based on whether the reaction qualified as whistleblowing, in order to separate this kind of reaction from other types of attempt at handling the situation. See the results in the table below. The “characteristic responses” in the table were selected to make it easier for the reader to follow what kinds of cases were coded in which category. [Table 4]

### Whistleblowing and consequences

The formalization of the duty to reporting misconduct is often justified with reference to consequences. Those who responded to the qualitative questions in the survey were concerned with consequences as well, but many of those who chose not to take action when discovering misconduct pointed to the potential negative consequences as reasons for their inaction. In the qualitative data, twelve of those opting not to take action gave the reason that they feared personal consequences. The following case illustrate most of the types of reasons the respondents gave. The respondent in the case feared that she would not be able to finish her PhD if deciding to take action, responding, “there is no going against my boss. There have even been lawsuits in the past but the University has always covered for her”. The perceived perpetrator of the misconduct had a managerial position, and the respondent reported that she had been involved in several forms of questionable practices for years, like self-plagiarism, undue authorships and unspecified questionable decisions in fieldwork.

For the respondent the combination of perceived high stakes, the potential loss of PhD-position, and very low chances of success due to lack of institutional support, counted as sufficient reasons for non-action. This case shows us that reporting misconduct can be perceived to be very difficult, especially if you are in a temporary position and the leadership at the institution is ready to defend the perceived perpetrator. Some of the other cases in this category specified that career consequences were what they feared, while others did not specify which consequences they had in mind. Some of them explicitly tied their worries with hierarchical concerns and lack of institutional support as seen in the case described in detail.

Whether or not the kind of fear described above is justified is relevant to how we should think about researchers’ duties when discovering misconduct. The data gives some insight into what actually happens when researchers report misconduct. In the failure category, of the 27 cases where the respondent reported the case, 18 ended with some form of negative outcome for somebody involved in the reporting. As mentioned, the data does unfortunately not give good insight into whether researchers are justified in being concerned for their careers, as researchers who lose their positions in such processes are unlikely to have received an invitation to answer the survey.

There are a couple of cases in the data however, where researchers left their jobs both voluntarily and involuntarily, after reporting misconduct. They were able to reply to the survey themselves in a few cases, as they had gotten new positions at other institutions, or had retained their institutional email in other ways. Sometimes more than one researcher is involved in handling the situation, and in one such case, the respondent stated that the primary victim of the misconduct lost her job.

While the data does not give us much insight into the most dramatic potential and feared consequences of reporting, it documents several other adverse consequences. In a case coded in the ambiguous category for example, the respondent had reported an undue change of authorship in a publication to the university committee for scientific integrity. This had the following result according to the responded “Good hearing, but decision unbalanced. Confidentiality violated by management, mistreated and reporting to the committee publicly portrayed as misconduct”. In addition, the respondent believed that “this may cost me my job”. She seems to have gotten some rectification here, but at a high price. The decision to report lead to mistreatment and counteraccusations, and she felt that the system was unable to secure her position.

Other cases involving negative consequences had varying degrees of severity. Some consequences were more informal, like harassment, severe emotional strain and conflict with colleagues. Others involved reactions of a more formal character, like counteraccusations and threats towards career prospects. As mentioned, some suffered consequences due to lack of rectification of the misconduct. This usually happened when the respondent was the victim of the misconduct, where the most common situation was undue denial of authorship, but also included theft of ideas and data.

The data shows that severe consequences happen to some of those who report misconduct and give us insight into what form this can take. The case described in detail, show us an example where the participant felt that research organizations can respond inadequately when researchers report misconduct, and this was a theme in many of the other cases as well, indicated by the fact that the respective research organizations were unable to protect those who reported from the negative consequences listed above. This qualitative data is, however, not well suited to tell us how prevalent such consequences or lackluster responses are. I will return to the question of risk and prevalence in the discussion section.

### Alternatives to reporting

The qualitative responses revealed approaches to handling the discovery of misconduct, other than reporting the situation. The most common attempt at social control after whistleblowing was direct confrontation of the perceived wrongdoer(s). Rather than reporting the case, some researchers attempted to rectify the situation themselves directly. In the data, this led to different results. Some succeeded where the situation was rectified. In one of these cases, the respondent, the editor of a journal, discovered “Several cases of self-plagiarism, but of a fairly innocent kind (paragraphs about methods)”. The respondent confronted the authors in question, and the issue resolved to his satisfaction when they reworked the problematic paragraphs. The respondent wrote that he had confidence that they would not do it again, but took a precaution nonetheless, by noting the cases in the journal’s database.

Some confrontations took the form of discussions, where the respondents tried to convince the perceived wrongdoers that what they were doing was wrong. In one case, the respondent tried to convince a colleague that he or she should stop “running multiple analyses and selectively reporting the significant ones”, and report findings more transparently. As she lacked good evidence, and was in a vulnerable position in the hierarchy, she decided not to report the situation. Several of the respondents chose to confront the perceived wrongdoer in this way, but found that reporting the case, thus blowing the whistle, had too high a threshold. These respondents were comfortable enough with exposing to the perceived wrongdoer that they were concerned, in an attempt at rectifying the situation, but if this effort failed, in some cases the respondents stated that this was as far as they were willing to go, due to fear of personal costs.

In several of the cases, the respondent had adopted the case from another researcher, and in some of these cases, they give the reason that this was because the one who actually discovered the misconduct felt too powerless to handle the case alone. In one case for example, the respondent had adopted the case of a colleague who was unduly excluded from authorship. The respondent confronted the perceived wrongdoers and “talked to them separately on the initiative of the “offended” party”. In the case descriptions, the respondents often state that they approach colleagues or other people they trust and discuss what can be done. They try to mobilize support, and sometimes they end up handing over or sharing the responsibility of trying to rectify the situation. In some cases, lack of such support resulted in the respondent dropping the case. In one example of this, the respondent learned about a case of self-plagiarism from a colleague. There was a discussion about it, but “There was no clear opinion among colleagues whether it was a misconduct or not so it was [not] reported”.

Another relevant phenomenon in the data was the researchers’ behavior after deciding whether to try to rectify the situation or not. The respondents found ways of protecting and promoting integrity after the fact, where they learn to be more careful in the future, for example protecting their ideas from plagiarism or other forms of theft. One respondent had experienced two instances of more senior researchers unduly demanding authorship on papers where she was the corresponding author. One of the senior researchers also “claimed to have partially come up with the idea (which was not the case)”. In one of the instances the respondent “gave in to avoid further repercussions”, and in the other case she let it slide to avoid another fight. In order to make sure that she would not find herself in such a situation again, she stated that “I am not collaborating with one of [them] anymore and I will try to keep better track of my study ideas”. In this way, she attempted to prevent further misconduct through documentation and breaking off relations with one of the senior researchers.

Other cases involved learning to create better contracts for further collaborations. In one case, an industry partner in commissioned research stopped the respondent from publishing their findings. While the respondent was unable to solve this situation, she learned that she should make better contracts in the future, which would ensure that she would be able to publish the findings. She also stated that her “mind and desire to collaborate with industry” had changed because of the situation.

Teaching other researchers how to avoid ending up in similar situations, based on what one has learned, is a possible way to prevent further misconduct. One respondent discovering that a reviewer had blocked a paper only to publish a very similar paper his or herself. He tried to confront the perceived perpetrator, but was only a graduate student at the time, and the result was that “they called me an idiot (I was a beginning PhD)”. He did not report the matter formally. Now however, the respondent is using this story as an example when teaching ethics.

The most dramatic response to discovering misconduct in the replies to the survey was leaving the research institution in question or leaving academia altogether. One researcher left academia after being involved in writing a paper where the two other authors engaged in “Unjustified and incorrect tuning of statistical analysis parameters in order to make significant results appear”. After trying to rectify the situation through confrontation and trying to mobilize support, the respondent writes “I left academia because I’ve had enough. This situation has been going on for years, and I’m not able to change it”. These actions may not solve the problem, but the respondents protect themselves by leaving a situation where they risk implication in misconduct.

## Discussion

### Should reporting misconduct be mandatory?

The data shows that researchers can find themselves in some very difficult situations when they discover misconduct. These findings are relevant for the question of whether reporting misconduct should be considered a duty, and whether we should formalize it in policy documents like codes of conduct. Those who promote including a duty to report research misconduct in codes of conduct typically argue, as we have seen, that the positive consequences of reporting outweigh the potential negative ones.

The respondents who chose not to report the perceived misconduct in the data presented in this paper tended to disagree. They believed that it would likely be without results, and/or that it would probably have significant negative consequences for themselves. They felt, among other things, that they lacked the necessary collegial support, that the university would protect the perpetrator, and that their jobs were at risk. The quantitative data shows similar attitudes. The respondents commonly feared that their universities would not take them seriously or protect them if they report misconduct. This distrust of institutions and the risks the respondents perceived in the cases gives us reason to worry that making reporting misconduct a formalized duty is too demanding.

In order to settle that question however, it is not enough to discuss the risks the respondents perceive. We must also ask whether their fears are justified. It matters whether they actually risk suffering negative consequences and whether their chances for success actually are low. If their fears are exaggerated, and they could in fact realistically rectify the situation safely, they can be criticized for not properly assessing the risks involved.

Some of the respondents experienced that reporting misconduct can have serious negative consequences, including mistreatment, counteraccusations, emotional trauma, retribution and loss of position. The question is whether these kinds of consequences are prevalent enough to justify the kind of risk evaluations that makes researchers refrain from reporting. Others who have studied the same phenomenon have concluded that reporting misconduct both inside and outside of academia involves serious personal risk (Freckelton 2016). According to one study in academia (Lubalin and Matheson 1999), 68% of the whistleblowers surveyed experienced negative consequences, where this in 23.6% of the cases took the form of loss of position, either through firing or non-renewal of contracts. Based on both the data included in this paper and findings from other studies, we can conclude that researchers have good reasons to believe that reporting misconduct is risky. Negative consequences are prevalent, and protection of whistleblowers is lacking.

What should we expect of a researcher in such difficult situations then? Should we create codes of conduct demanding that they report the misconduct they discover? Those who support this view argue that reporting misconduct is important, because this prevents the corruption of the academic culture, protects patients from potential harm and prevents waste of resources, among other things. This outweighs the potential discomfort of reporting, the argument goes.

This is a good argument only if researchers have a reasonable chance to achieve these consequences. The respondents state however, that they believe that the chances for success were very low to non-existent, as they perceive significant institutional and collegial barriers to handling the situation in this way. If the consequences are what we care about, it seems that researchers can find that the potential consequences are worse than the potential positive outcomes. According to Jubb, “Whistleblowing is about stopping, hindering or preventing perceived wrongdoing. A disclosure method that has negligible prospect of achieving this result is self-destructive folly, not whistleblowing” (Jubb 1999, 88). I agree, and in the data and in the literature on whistleblowing, too many researchers find themselves in situations where they feel that reporting misconduct will have significant negative consequences for themselves, with little chance of rectifying the situation, where the consequences of blowing the whistle is worse than doing nothing. Research on what happens to whistleblowers give them good reasons to feel this way. The expected consequences are therefore significant sacrifices, with only a slim chance of success, a self-destructive behavior that would not be worth the risk and should therefore not be made mandatory.

In the ethics literature, discussions regarding whether ethical demands are too strict are discussed under the rubric *demandingness* (McElwee 2017)*.* These discussions typically consist in criticisms of consequentialist thinking, which at times can demand great personal sacrifices in order to bring about the best consequences. As argued above however, mandating that researchers report misconduct is too demanding even for consequentialists, as it will not reliably bring about the best consequences. Some argue that certain good actions should be considered *supererogatory* (Beauchamp and Childress 2009). This means that while we have certain duties, other good acts go beyond our duties, and people can do more than what is required as an ethical minimum. In the case of whistleblowing, this can involve voluntarily accepting the risk of reporting, even though this is not required either in codes of conduct or from an ethical point of view. Under this view, while reporting misconduct should not be considered a duty in difficult cases, researchers can accept the risk of blowing the whistle, and this would be a praiseworthy act that go beyond their minimal duties.

While we should make room for supererogatory acts in whistleblowing, we should also be concerned with the dangers they involve. According to Swanton, supererogatory acts are in general appropriate when they are “… effective, not damaging (or excessively so), and may be stepping stones to greater strength in the agent” (2003, 211). Conversely, they are less appropriate if they are inefficient, excessively damaging, or not a good source of learning. Attempts at supererogatory acts can fail at achieving their goals, and you can do more harm than good if you overreach and try to be “virtuous beyond your strength” (Swanton 2003, 211). If we return to the cases in the data where the researchers felt that reporting would be inefficient and involve significant risks to themselves, we can say that taking action in such a case would be supererogatory and praiseworthy in showing a willingness to make sacrifices in the name of trying to preserve the integrity of research. At the same time however, if the researchers are right that reporting would be inefficient and harmful, reporting can also do more harm than good and be self-destructive, which would detract from the appropriateness of blowing the whistle. Swanton argues that the principle that one should not overreach when trying to do good should be considered a warning and not a universal requirement. Applied in the context of whistleblowing this means that we should encourage caution when somebody is contemplating whether to report misconduct under high risk circumstances, while at the same time appreciating their effort if they go through with it.

An important aspect I have not yet discussed here is the nature of the misconduct, and its potential to have harmful consequences for third parties. Research fraud and misconduct sometimes leads to people dying or getting hurt in other ways (Vaux 2016). Some argue that in cases where there is a risk of immediate harm towards humans or animals blowing the whistle should be mandatory and formalized in codes of conduct (see for example Redman and Caplan 2015). Under this compromise, reporting misconduct is considered voluntary and supererogatory in cases with less severe consequences for third parties, like plagiarism and undue distribution of authorship. When the misconduct in question can have serious negative consequences for third parties however, like falsification of data in clinical trials, reporting misconduct goes from being voluntary and supererogatory to being mandatory, according to this argument.

Consequences have played an important role in the arguments made so far in this paper, and the potential negative consequences for third parties should be something researchers take into account when they discover misconduct and consider whether to blow the whistle. Accepting this view makes it tempting to try to establish some criteria for how researchers should make these decisions. How much risk should they be willing to accept? How should they weigh various consequences against each other? Is there a threshold where the negative consequences of the misconduct are so serious that we should move from considering whistleblowing as supererogatory to considering it as mandatory, and should such a threshold be introduced in codes of conduct? However, asking these questions masks relevant ethical aspects of such situations. There is more going on than a binary decision about whether to report the case or not. Rather than trying to settle what an appropriate threshold for when one should report misconduct would be, I will argue in the next section that we need a broader perspective on the topic.

### A broader perspective on duties when discovering misconduct

Considering the cases through a social control perspective revealed a broader spectrum of reactions to discovering misconduct than just reporting and whistleblowing. These reactions can be divided into two categories, alternatives to reporting, and promoting integrity after the fact. As the cases show, discovering such conduct does not necessarily put one in a position where one must make a binary decision either resulting in reporting or a decision not to do so. Discovering misconduct does not lead to a dilemma, but to a process where there can be several different possible approaches to handling the situation, and where there are ethically relevant things one can do after deciding whether to report or not. Asking whether researchers should report misconduct or not faces what Appiah has called a *packaging problem*. As he writes,

In the real world, situations are not bundled together with options. In the real world, the act of framing–the act of describing a situation, and thus of determining that there’s a decision to be made is itself a moral task. It’s often *the* moral task (Appiah 2008, 196).

Ethics is, in the real world, often more about understanding and navigating complex situations, identifying relevant options, than it is about picking the correct answer to dilemmas.

In the cases, the researchers often start by exploring their options and try to mobilize support when discovering misconduct. They evaluate the risks involved and proceed step-by-step, until they reach their risk tolerance. If they decide to take action, this can for example take the form of direct confrontation, which can be a way to “nip misconduct in the bud” (Koocher and Keith-Spiegel 2010). In some cases where the respondents lacked good evidence, or felt that they were in vulnerable positions, they chose to try to discuss the case with the perceived wrongdoers and negotiate some solution. Confrontation, discussion and negotiation can therefore be a substitute for reporting when researchers believe that reporting is too difficult. Another alternative was handing the case over to somebody in a better position to act. When researchers feel that they are in a situation where reporting is too risky, they can talk to their colleagues and strengthen their position. This can lead to others taking responsibility for handling the situation, and shows that reacting to misconduct can take the form of a social process, even though it is often construed in the literature as a dilemma with a solitary actor.

The alternative to reporting is therefore not necessarily non-action, and researchers are therefore not off the hook if they find that reporting is too risky or that it is pointless. As Appiah points out, they can look for options, and this paper shows that such options exist. The question we should be concerned with therefore, is not “should researchers report misconduct when they discover it?” but rather “how can researchers best preserve the integrity of research when they discover misconduct, within their capacities in the situation in question?” By formulating the question about researchers’ duties in this way, we keep it open what actions they should take, and we acknowledge that the organizational context, risk and their capacities to successfully deal with the situation are relevant factors for how we should think about their duties.

Formulating the problem in this way also opens a discussion about what researchers should do after the fact. Preserving integrity when discovering misconduct is not limited to attempting to handle the situation in question directly. In the data, there are examples of how researchers learn from their experiences and engage in preventative behavior, by making misconduct harder or teaching others to avoid it. Vaidyanathan et al. have documented a similar phenomenon, based on their observation that researchers can use gossip as a substitute for reporting misconduct (Vaidyanathan et al. 2016). If they feel that reporting the misconduct is beyond their risk tolerance, they warn other researchers, and exert social control through gossiping about it.

In the data, the respondents engaged in several ways of improving the integrity of research, after deciding whether to report the situation or not. They describe things like improving idea protection to prevent theft, restructuring networks to avoid having to collaborate with perceived wrongdoers, creating better contracts for commissioned research in order to secure control over how the results are communicated, and using their experiences to teach others how to avoid similar situations.

This behavior aims at preventing further misconduct, and therefore promotes integrity and constitutes a form of self-regulation. As this kind of behavior can promote integrity after one discovers misconduct, it falls within one’s duties in such situations. If the discussion is limited to the question of whether researchers should report misconduct or not, the duties researchers have after the fact are masked. Even researchers who decide to blow the whistle still should learn from the experience and work towards preventing further misconduct. This is especially true in cases where they failed to rectify the situation, as their experiences with the difficulties of handling the case can be useful for others.

When trying to determine whether researchers did the right thing when discovering misconduct, we should not limit ourselves to asking whether they reported the situation. Researchers deserve a more holistic ethical evaluation. If we want to know whether researchers handled discovering misconduct in a good way, it is relevant whether they thoroughly evaluated the options that were available, whether they attempted to solve the situation through other means than reporting, and whether they took action after the fact to ensure that such situations would not happen again. Even those who believe that reporting misconduct should be mandatory should accept that trying to solve the situation in some other way, or attempting to prevent further misconduct, is better than doing nothing, and that this is therefore ethically relevant.

A social control perspective on how researchers react to discovering misconduct, can be helpful in discussions about how research integrity can be promoted in research organizations. The complexities of situations researchers can find themselves in when discovering misconduct should be taken into account in research integrity training, for example in the form of discussion of the kind of difficult cases included in this paper. Researchers should be taught that deciding whether to report misconduct or not is not an ethical dilemma, with a yes or no answer, but that it is also about mapping options, mobilizing support and taking preventative action so that one avoids such situations in the first places. Leaders in research could benefit from insight into how researchers react to discovering misconduct, and what their worries are, so that they can make the process towards rectifying such situations smoother.

It is worth underscoring that the researchers in the data tended to seek out alternatives to reporting misconduct due to deficiencies in the systems where they find themselves. An important lesson from this and other research on how researchers react to discovering misconduct is that research organizations should take steps to ensure that reporting misconduct is safe and reliable. An ideal whistleblowing system has merits that should make it the preferred way to deal with misconduct. When it works, it protects both the accused and the accuser (Bouter and Hendrix 2017; ALLEA 2017; Forsberg et al. 2018), and secures a speedy and impartial investigation. Until such a system is in place however, we should be careful about condemning researchers for not reporting, as the risks involved limits their duties.

When it comes to codes of conduct, the Norwegian *Guideline for Research Ethics in science and Technology* published by *the Norwegian National Research Ethics Committees*^7^ promotes the type of view I have argued for and can serve as inspiration for others. These guidelines state that whether potential whistleblowers should report misconduct depends in part upon the circumstances, including the potential risks towards the researchers’ own interests and the potential negative consequences of the misconduct. The guidelines also cover the responsibilities of research organizations.

### Concluding remarks

The duties described above can be summarized in three points:

1. Researchers have a duty to take action when discovering research misconduct, within their capacities in the situation in question. If the potential negative consequences of the misconduct in question are severe, their risk-tolerance should increase proportionally
2. Discovering misconduct should be a teachable moment, and the experience should be used for preventing further misconduct
3. Institutions have a duty to make reporting safe and efficient, and the failure of institutions to ensure the safety of whistleblowers, limit the duties potential whistleblowers have

By formulating researchers’ duties in this way, we keep it open what approach the researcher in question can choose when it comes to taking action. As we have seen, it can be too demanding to make it mandatory for researchers to report misconduct, but there can be options, other forms of social control, that are within the capacities of the researcher, and these should be pursued. Researchers should also contribute to a climate of integrity by learning from discoveries of misconduct. They should use their experience to teach others how to avoid finding themselves in a similar situation, and they should try to prevent it from happening again in the further.

When we frame the duties in this way, we also make it a question about institutional responsibility. The strengths and capacities of researchers, and the situations they find themselves in, are to an extent the responsibility of their employers and managers. They are responsible for training their employees and making sure that it is safe to raise concerns when discovering misconduct. If we make it a formalized duty to report misconduct, we risk putting researchers in an impossible situation, where they would have to choose between suffering serious personal consequences for a slim chance at rectifying the situation, and making themselves guilty of misconduct themselves by not taking action and breaking the codes of conduct. It would be more prudent to hold the institution responsible first, until the situation is such that it is safe enough to take action. We should only criticize inaction if it is within the capacities of the researcher in question to reliably rectify the situation, or if the misconduct in question is likely to cause serious harm.

## Data Availability

The data is deposited at the Norwegian Center for Research Data. It can be made available on request to the author.

## Footnotes

1 They also write that this does not have to take the form of *full-scale whistleblowing*. Unfortunately they do not provide clear definitions of *raising a concern*, *reporting* and *whistleblowing*, so it is difficult to evaluate exactly where they place the threshold for what it takes to fulfill one’s duties when discovering misconduct

2 PRINTEGER (*Promoting Integrity as an Integral Dimension of Excellence in Research*) is a Horizon 2020 research project – www.printeger.eu

3 The research protocol was published as a deliverable in the PRINTEGER-project, and is available here: https://printeger.eu/wp-content/uploads/2018/06/4.1-research-protocol-for-questionnaires-focus-groups.pdf

4 Two additional papers are underway based on the survey. One analyzes the quantitative parts of the survey, while the other uses descriptive statistics to look at demographics and other variables when it comes to how disposed researchers are towards engage in whistleblowing

5 The PRINTEGER-deliverable referenced in this section gives further insight into the sample as compared with the population

6 Some were unclear (*n* = 3), about an instance already reported and handled by others (*n* = 12), about student misconduct (n = 3), the respondent was the guilty party (n = 1) or the respondent had only heard about the case (*n* = 9). In addition, some of the cases were still unresolved (*n* = 11) and some had an unclear result (*n* = 5), and are therefore disregarded in this analysis. Some cases where students were involved are included in the analysis. These were included when the students were involved in research activities, or were the victims of abuse by researchers

7 https://www.etikkom.no/en/ethical-guidelines-for-research/guidelines-for-research-ethics-in-science-and-technology/whistleblowing-and-ethical-responsibility/
